# Thermal engineering of stone increased prehistoric toolmaking skill

**DOI:** 10.1038/s41598-019-51139-3

**Published:** 2019-10-10

**Authors:** Veronica Mraz, Mike Fisch, Metin I. Eren, C. Owen Lovejoy, Briggs Buchanan

**Affiliations:** 1 Department of Anthropology, University of Tulsa, Tulsa, Oklahoma 74104 USA; 2 College of Aeronautics and Engineering, Kent State University, Kent, Ohio 44242 USA; 3 Department of Anthropology, Kent State University, Kent, Ohio 44242 USA; 4 Department of Archaeology, Cleveland Museum of Natural History, Cleveland, Ohio 44106 USA

**Keywords:** Materials science, Archaeology

## Abstract

Intentional heat treating of toolstone has been documented to have begun at least by 70 K BP; however, the advantages of such treatment have been debated for decades. There are two schools of thought with regard to its purpose. One, is that it merely reduces the force required for flake propagation. A second is that it also alters flake morphological properties. We systematically tested these hypotheses by generating flakes from cores exposed to three different temperatures (ambient, 300 °C, and 350 °C) using automated propagation procedures that bypassed any human agency. While the force propagation magnitude is altered by heat treatment, the flakes were not. We examined these flakes according to nine measures of morphology. None differed significantly or systematically within the three categories. While our results confirm that heat treatment does reduce the force needed for flake propagation, they also demonstrate that such treatment has no significant effect on major morphological aspects of flake form.

## Introduction

Archaeologists have long argued that heat treatment improves the quality and workability of toolstone^1–8^. Heat treatment (HT) usually involves slowly heating stones to approximately 200 °C to 500 °C^9^, but other strategies have been explored as well (e.g.^10^). The earliest documented case of intentional and systematic HT is from the South African Middle Stone Age, over 72 thousand years ago^11–13^. Since that time, lithic HT has been documented archaeologically and ethnographically throughout the world (e.g.^12,14^).

The mineralogical, chemical, and crystallographic effects of relatively high heat applied to stone have been examined thoroughly (e.g^13,15–22^.). However, Schmidt *et al*.’s^14^ critical review has highlighted a current gap in our understanding of its effect on stone fracture mechanics. This is critical because stone’s “workability” may have been the potential conscious or subconscious primary target of HT by earlier humans. Indeed, as Wadley and Prinsloo^13^ and Schmidt *et al*.^14^ outline, HT has been linked to the evolution of complex human behavior and cognition, technical skill, and specialized craftsmanship^11,23–31^.

Heat treatment has been argued to improve the workability of stone for knapping essentially in two ways. The first is that it purportedly reduces the force magnitudes needed to initiate fracture^17,18,32–36^. This has been robustly supported by direct observation via materials testing, as well as by quantitative assessments of force required to detach flakes^11,12,14,37–40^. The second is that it may alter the size and shape of flakes removed from a core^41–45^. For example, Bleed and Meier^33^ have suggested that heat-treated flakes are larger and longer than flakes removed from “raw” (i.e., untreated) cores. Likewise, Cooper^46^ has suggested that heat-treated stone yields larger flakes than its untreated counterpart, while Hanckel^47^ has opined that flakes produced from heat-treated stone are thinner than are those removed from untreated material. Moreover, Collins and Fenwick^48^ have suggested that heat-treated flakes are less variable in form than are raw ones, as they purportedly have fewer step and hinge terminations. Flenniken and Garrison^49^ also assert that a knapper has potentially more control of the form of flakes removed from a HT core than from a “raw” one. Finally, Rick and Chappell^45^ have argued that heat-treated flakes are sharper than their raw counterparts because they putatively bear more acute edge angles than do flakes derived from unheated cores.

Following Eren *et al*.^50^, one can argue that HT interacts with other input variables in two different hypothetical ways, which constitute polar opposites. The first or “natural forces” hypothesis^51,52^ posits that artifact morphology depends exclusively on whether core stone is raw or heat treated when knapped. A corollary is that behavioral and cultural factors play no role in determining artifact form, and that natural stone constrains or “dictates” artifact morphology. The contrary view is represented by the “artificial forces” hypothesis^51,52^. This holds that HT has no substantial effect on artifact morphology, which instead lies only with its knappers. This hypothesis holds that there is nothing inherently different about raw versus heat-treated rock types that can influence artifact morphology–artifact form ultimately lies only with knappers themselves.

One way to assess these two hypotheses is to perform a flintknapping experiment in which human influence has been removed from flake production^53–56^. Previous experimenters have attempted to do this, but with only limited results. For example, Bleed and Meier’s^33^ experiment rolled raw and heat-treated stone tiles in a mechanical drum. However, this mimics geologic processes more closely than flintknapping. Cooper^46^ dropped ball bearings on slabs of raw and heat-treated stone, but he examined only ten samples of each, and reassessment of his flake morphology data contradicts his conclusion that HT increases flake size (see Cooper ^46^:Table 1).

**Table 1:** Results of Shapiro-Wilk tests for normality for the seven variables measuring the experimentally-produced flakes from the three core groups.

Variable	W	P
Weight	0.9792	0.1594
Length	0.9767	0.1048
Width	0.9829	0.287
Width at 25% length	0.9736	0.064
Width at 50% length	0.9736	0.1521
Width at 75% length	0.9813	0.2231
Thickness	0.4884	<0.0001*

*Benjamini and Yekutieli^73^ adjusted significance level for 7 tests is 0.01928.

To overcome these limitations, we produced flakes from raw and two types of heat-treated cores using an Instron Universal Materials tester, and then compared their form within three datasets as defined by the temperatures of their heat treatment. Of major import is our ability to determine the magnitude of the force required to initiate flake fracture independent of any human agency other than HT itself. Our experiment has allowed us to demonstrate that HT cores exhibit less resistance to fracture propagation (i.e, less energy is required to generate fracture and they are thus less tough than are raw cores). This has allowed us to examine whether toughness alone consistently leads to different flake forms, which in turn has provided us an opportunity to reliably test the natural versus artificial force hypotheses.

## Results

**Figure 1:**
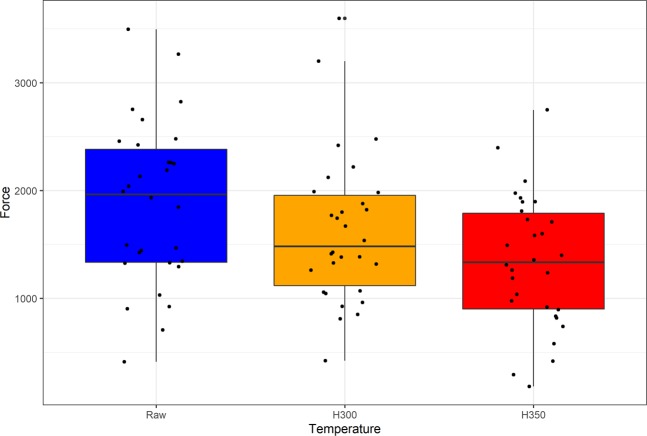
Boxplots of the force needed to detach experimental flakes by temperature (from raw cores [blue box], cores heated to 300 °C [orange box], and cores heated to 350 °C [red box]). The bars indicate the median, the boxes show the upper and lower quartiles of the data distribution, and the whiskers show the distributions without outliers. The data are shown as black circles.

We first examined the force magnitudes required to detach flakes in each of the three test groups. Their distributions do not significantly differ from an underlying normal distribution (Shapiro-Wilk test W = 0.99, p = 0.7396). Boxplots show that the force magnitudes required to detach flakes from the raw (ambient) cores appear to be greater than those required to remove flakes from cores heated either to 300 °C or to 350 °C (Fig. 1). The ANOVA of the force data by core group supports this observation (F = 4.522, df = 2,87, p = 0.0135). Multiple comparisons using Tukey’s HSD method indicate that the force needed to remove flakes from raw cores is significantly greater than the force required to detach flakes from cores heated to 350 °C (difference 535.33, p = 0.009), but the force used to remove flakes from raw cores and cores heated only to 300 °C did not differ significantly (difference 249.08, p = 0.3464). The forces required to remove flakes from cores heated to 300 °C and 350 °C also do not differ significantly (difference 286.25, p = 0.2483).

**Figure 2:**
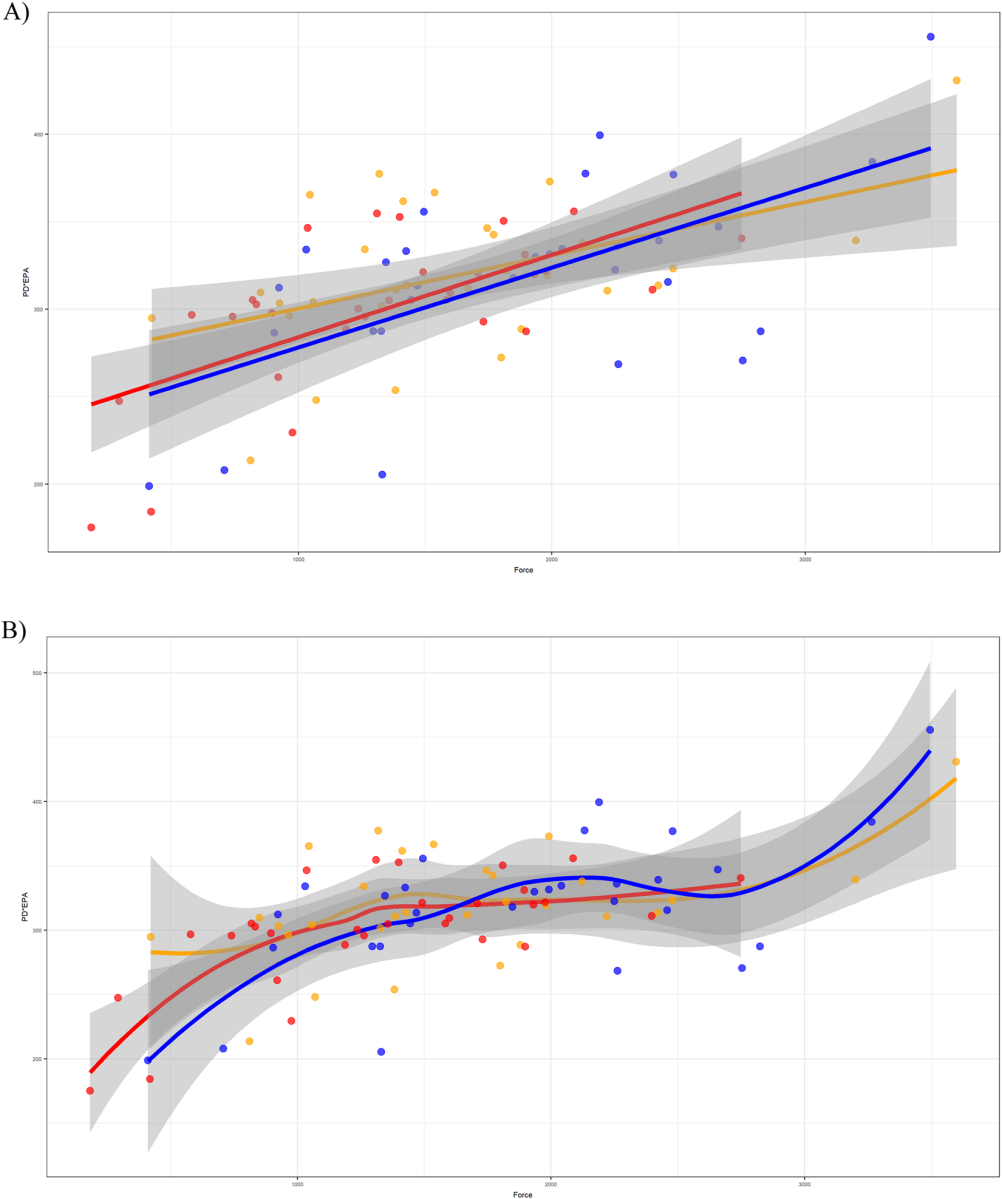
Plot of the ANCOVA results showing (**a**) linear fit lines, and (**b**) smoothed lines modelling the relationship between the interaction between exterior platform angle and platform depth and force by core group (flakes derived from raw cores are shown as blue circles, flakes derived from cores heated to 300 °C are orange circles, and flakes derived from cores heated to 350 °C are red circles).

We next added platform depth and exterior platform angle as variables in our analysis of force magnitude, as both variables have been reported to affect flake size^57–63^. It is therefore also likely that they affect the magnitude of force required to detach flakes. The sample distributions of platform depth (W = 0.96, p = 0.0046) and exterior platform angle (W = 0.97, p = 0.0447) differ significantly from an underlying normal one (exterior platform angle is only borderline significant). Transformations of the platform depth and exterior platform angle variables did not result in normality. Given this non-normality of some variables, we used a nonparametric ANCOVA to control for interaction between platform depth and exterior platform angle, while examining differences in mean force magnitude among the three core groups. Our results suggest a significant difference in force required to detach flakes among the three treatment groups (h = 18.93, p = 0.0337). Figure 2 shows the relationships among the three variables and core groups. The force needed to remove flakes from cores heated to 300 °C shows some interaction with the force needed to remove those from raw cores in both the linear (Fig. 2a) and smoothed (Fig. 2b) fit lines. The cores heated to 350 °C required less force to remove flakes after controlling for exterior platform angle and platform depth.

Following the analyses of force magnitude differences, we examined the potential differences in flake form amongst the three test groups. We first examined differences in flake form by temperature group without regard of the differences in force necessary to detach the flakes. We did this because we are interested in the morphology of the flakes given the same starting condition of initial detachment. Tests of normality indicate that only maximum thickness differs significantly from an underlying normal distribution (Table 1). Logarithmic and square root transformations of thickness measures did not reduce the skewness in the sample distribution. Accordingly, we carried out ANOVA tests for six of the variables (weight, length, maximum width, width at 25%, width at 50%, and width at 75%) and a Kruskal-Wallis nonparametric test for one variable (maximum thickness) by core group. The results indicate that none of the measurement variables differed among the core groups (Table 2) and that the Kruskal-Wallis test indicates that thickness also did not differ among core groups (K-W chi-square = 1.047, p = 0.5923). Additionally, Kruskal-Wallis tests of platform depth and exterior platform angle suggest no differences among core types (Platform depth: K-W chi-square = 2.804, p = 0.2462; Exterior platform angle: K-W chi-square = 0.8922, p = 0.6401).

**Table 2:** ANOVA results for the six variables measuring the experimentally-produced flakes from the three core groups.

Variable	F	P
Weight	0.122	0.885
Length	1.285	0.282
Width	0.493	0.613
Width at 25% length	1.553	0.218
Width at 50% length	0.442	0.644
Width at 75% length	0.224	0.800

Lastly, we compared the form of flakes by temperature group using a general linear modeling approach to control for force, exterior platform angle, and platform depth. The results of these analyses demonstrate that flake form does not differ across temperature group when force, exterior platform depth, and platform depth are included in the models (GLM results presented in the Supplementary Materials). We conducted further analyses of the seven flake variables using nonparametric ANCOVAs with force and platform depth and force and exterior platform angle as covariates. The results show that none of the variables are significant among the temperature groups (Table 3). Thus, there are no morphological differences among the flakes by temperature group when force, platform depth, and exterior platform angle are considered simultaneously.

**Table 3:** Results of nonparametric analysis of covariance with force magnitude and platform depth and force magnitude and exterior platform angle by temperature group.

Covariates = Force + Platform depth	p-value	Covariates = Force + Exterior platform angle	p-value
Weight~Temperature	0.942	Weight~Temperature	0.6773
Length~Temperature	0.7086	Length~Temperature	0.5376
Width~Temperature	0.9875	Width~Temperature	0.6852
Width at 25%~Temperature	0.9362	Width at 25%~Temperature	0.8483
Width at 50%~Temperature	0.9868	Width at 50%~Temperature	0.7736
Width at 75%~Temperature	0.9499	Width at 75%~Temperature	0.6357
Thickness~Temperature	0.9704	Thickness~Temperature	0.9996

## Discussion

The heat treatment of stone is a potentially transformative procedure commonly thought to be among the earliest human efforts to alter the properties of naturally available materials^11,13,14,64^. Several studies, including this one, have demonstrated that HT reduces the amount of force necessary to initiate flake fracture from a core. Yet the influence of this transformation on stone flake morphology has, until now, remained unknown.

Our experimental results showed no significant difference between the forms of raw versus heat-treated stone flakes both with and without the covariates of force magnitude, platform depth, and exterior platform angle. This suggests that HT does not alter flake form. Or, more precisely, HT does not render stone more “functional”^28,65^. Both HT and “raw” stone yielded virtually identical flake forms. Therefore, any failure to obtain a desired product lies merely with a knapper’s knowledge, manual dexterity, and skill. As such HT merely serves as a “process control”, which Patten^66^ has defined as a systematic modification of a process that augments a knapper’s skill. Heat treatment certainly adds time and energy to knapping, but by reducing a stones’ resistance to fracture propagation^14^ it augments a knapper’s skill by lowering arm acceleration, and thus the force needed to generate a crack. This, in turn, allows him or her to focus on the precision and accuracy of blows. That is, HT facilitates the knapping process, but does not alter or potentially improve the flakes it produces. Thus, our findings reject the “natural forces” hypothesis and support the “artificial forces” hypothesis. Artifact morphology lies only with a knapper’s skill and knowledge.

Finally, our finding that force magnitude has no impact on flake form is supported by other experiments. Controlled experiments using glass have shown similar results so long as other independent variables such as exterior platform angle, platform depth, and angle of blow are held constant^59^. In other words, these three variables dictate flake form even when they are produced by significantly different magnitudes of fracture initiation force. When our results are considered in light of previous controlled knapping experiments, we can suggest more broadly that conchoidal fracture propagation is similar among different kinds of toolstones, although future tests should assess the universality of our heat-treatment results on non-chert toolstones like silcrete or quartzite. The only factor that differs is the amount of force needed to generate a crack. Thus, the reduction in force magnitude that HT renders does not appear to alter the primacy of other variables in stone flake formation; it simply allows knappers to focus on these variables during a strike. Heat treatment can enhance a knapper’s skill, but it does not “improve” the stone.

## Methods

We used 47 standardized chert cores in our experiments. All were of Keokuk, a white to gray chert with a medium-grained texture, but which can range in quality from coarse to fine with a dull luster^67,68^. Keokuk is deposited in discontinuous bedded planes of the Boone Formation that are exposed in southeastern Iowa, southwestern Missouri, northern Arkansas, and northeastern Oklahoma^69,70^. We used trapezoidal cores with average lengths of approximately 110 mm and breadths of 80 mm wide at the top and 20 mm wide at the bottom (see SOM). We heated 15 of these cores to 300 °C and 15 to 350 °C (see below). We also used 17 raw (ambient) cores. We obtained two flakes from each of the large faces of each core, except in two cases when only one flake each was detached from two raw cores. This produced a sample of 90 flakes, 30 in each temperature group: raw, 300 °C, and 350 °C. The cores had an exterior edge angle of approximately 65° (the exterior edge angle varied between 60° and 67°; this variation was due to the difficulty in cutting the cores precisely). We chose this target exterior edge angle in order to be consistent with previous experiments that found it to be effective for removing flakes^56,57,59,60,70^.

**Figure 3:**
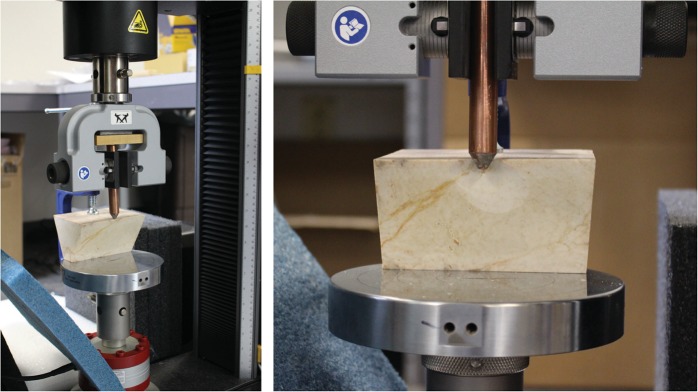
Photographs of the Instron Universal Testing Machine with Keokuk chert core clamped in place. (**A**) core clamped in Instron prior to flake removal with copper indentor, (**B**) close-up of core in the Instron after flake is detached with copper indentor.

We used a Paragon Sentry 2.0 kiln (programmed in Fahrenheit) to heat-treat two groups of cores (SOM). For the experimental flake removal we used an Instron Universal Materials Tester, which simultaneously recorded the force needed to detach each flake (Fig. 3, see also SOM). Prior to detaching each flake we inscribed a line 3 mm from the lateral edge of each core as a target for the indentor. As the indentor’s point was blunt we simply visually centered it on the inscribed line. We programmed the Instron to perform at a velocity of 1000 mm/min.

Following the protocol of Buchanan *et al*.^71^, we measured nine variables on each of the 90 flakes generated in our sample. These were as follows: platform depth, exterior platform angle, weight, length (measured from the platform orthogonally to the termination), maximum width (taken at the widest point orthogonal to the length measurement), width at 25% of length, width at 50% of length, width at 75% of length, and maximum thickness.

We separately analyzed two data sets that we obtained from the Instron and the resulting flakes from the Instron experiment. We first examined only the force magnitudes required to remove flakes from the three groups of cores (raw, 300 °C, and 350 °C). We then analyzed the form of removed flakes from each group using separate procedures.

For each analysis we assessed normality using Shapiro-Wilk tests^72^ and ran univariate parametric and, where appropriate, nonparametric ANOVAs by core group. For multiple tests we used Benjamini and Yekutieli’s^73^ (also see^74^) method to adjust the significance level. For our examination of the simple force magnitude experiment we followed our univariate tests with a nonparametric ANCOVA model design^75^ to test for differences in force magnitude needed to detach flakes analyzed by core group and also to examine any role of platform depth and exterior platform angle, as these variables are known to affect flake size^57–63^.

For our examination of flake morphology, we first conducted univariate comparisons of seven flake morphological variables and two flake platform characteristics by temperature group. Following the univariate analyses of the flake variables we used general linear modeling to evaluate the relative effects of each variable with force magnitude, platform depth, and exterior platform angle included as covariates. We then subjected the variables to nonparametric ANCOVA analyses as described above using force magnitude and platform depth as covariates in one set of analyses and force magnitude and exterior platform angle in the second set of analyses. We conducted all of our statistical analyses using R version 3.5.1^76^ and RStudio version 1.1.456^77^. The data and R script for analyses are provided in the Supplementary Materials.

## Supplementary information

Supplementary Materials

Data on experimental cores

Instron force and flake data

R script
