# Supplementary material for: Thermal engineering of stone increased prehistoric toolmaking skill
Source: Sci Rep. 2019 Oct 10;9:14591. doi: 10.1038/s41598-019-51139-3 (PMC6787202; doi:10.1038/s41598-019-51139-3)
Supplement: Supplementary file 1 — Supplementary Materials [file 41598_2019_51139_MOESM1_ESM.docx]

**Supplementary Materials for Mraz et al.**

**Thermal engineering of stone increased prehistoric toolmaking skill**

Veronica Mraz^1^, Mike Fisch^2^, Metin I. Eren^3,4^, C. Owen Lovejoy^3,4^, and Briggs Buchanan^1^*

1. Department of Anthropology, University of Tulsa, Tulsa, Oklahoma, 74104

2. College of Aeronautics and Engineering, Kent State University, Kent, Ohio, 44242

3. Department of Anthropology, Kent State University, Kent, Ohio, 44242

4. Department of Archaeology, Cleveland Museum of Natural History, Cleveland, Ohio, 44106

*To whom correspondence should be addressed: Briggs Buchanan, Department of Anthropology, University of Tulsa, Tulsa, Oklahoma, 74104, [briggs-buchanan@utulsa.edu](mailto:briggs-buchanan@utulsa.edu)

1. **Core design**

The Keokuk cores were cut to be trapezoidal in shape with an approximate exterior edge angle of 65° (the exterior edge angle generally various between 60° to 67° due to the difficulties in cutting chert). Initially, the cores were cut to be approximately 11.5cm long by 6.5cm high and 10cm wide (Fig. S1). The size and shape of the cores best suited to the laboratory experiments was determined using the results of a pilot study (see Mraz 2019). From the pilot study, it was determined that a longer trapezoidal core allowed the flake to widely propagate (as there was no ridge for the force to follow) without running into the sides of the core, impeding the width of the flake. The cores were cut by Craig Ratzat of Neolithics Quarry (Quapaw, Oklahoma). The cores were cut on oil cooled drip box saws; Mr. Ratzat used Highland Park model saws. The resulting cores weighed on average 972.64 grams (weights of individual cores are given in Table S1 Excel file).

65ᵒ

65ᵒ

4cm

10cm

11.5cm

6.5cm

Fig. S1. Design for core cutting.

1. **Core heating procedures**

To sufficiently dry each group of core to be heat treated, we first raised the kiln temperature to 93.33°C (200°F). For both modified groups we maintained this for 12 hours. We then elevated the kiln temperature to a second plateau of 273.89°C (525°F) at a rate of 22.22°C (40°F) /hour. We then further increased temperature to a third (and final) plateau of 300°C (572°F) at a rate of 5.56°C (10°F) /hour. This maximum was held for six hours. We then lowered kiln temperature to 273.89°C (525°F) at a rate of 15°F/hour. At this point the kiln was depowered and the cores allowed to cool passively until they reached room temperature. We heat-treated Group II identically, except the second plateau was set at 315.55°C (600°F) and the third was set to 350°C (662°F).

Our Instron 5967 has a 30kN static load cell with an accuracy of 1/1000^th^ of the force capacity. To detach flakes we used a 15cm diameter compression anvil as a base located immediately above the load cell and a copper rod with a conical blunted point as the indentor (see Figure X). We fashioned the latter from an 11cm long, 16mm diameter, grounding rod. The indentor was held in place by a side-screw action grip, and each core were secured to the compression plate using a 4in C-clamp.

To record variables on our 90 flakes (Figs. S2, S3, and S4), we used Mitutoyo digital calipers for each metric rounded to the nearest 0.1 millimeter and an Ohaus Scout Pro 602 digital scale rounded to the nearest 0.01 gram to measure weight.


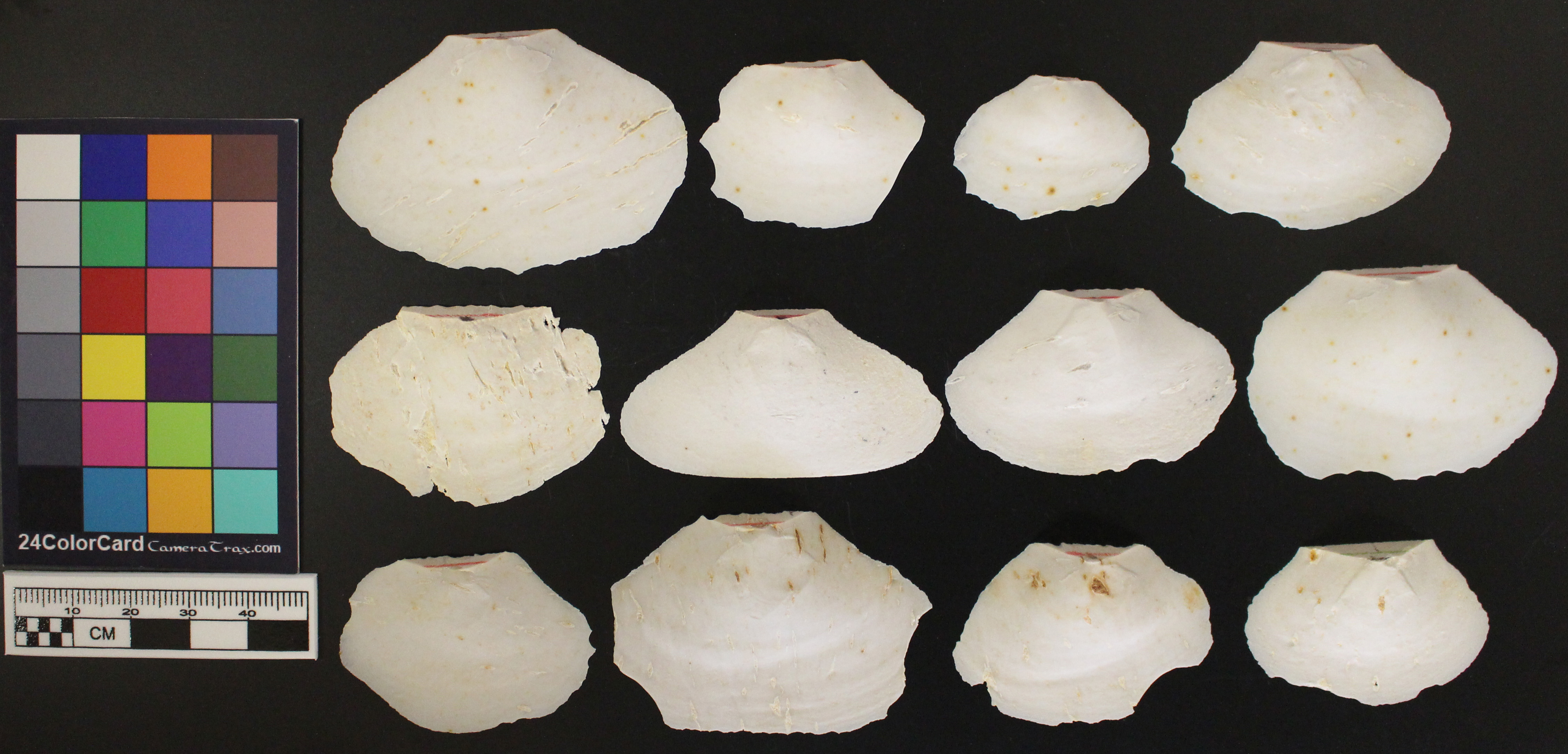


Fig. S2. Photograph of select flakes removed from unheated cores.


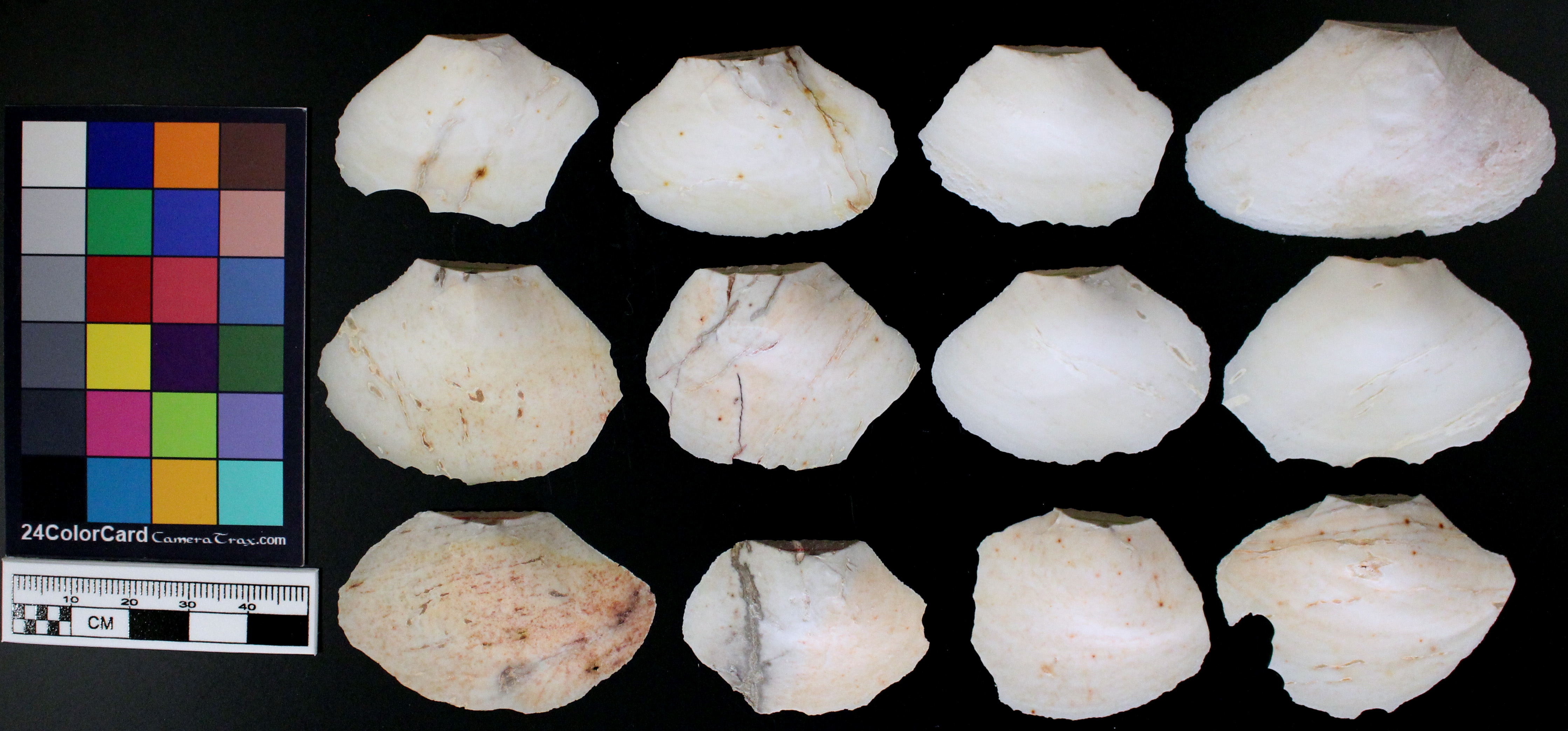


Fig. S3. Photograph of select flakes removed from cores heated to 300ºC.


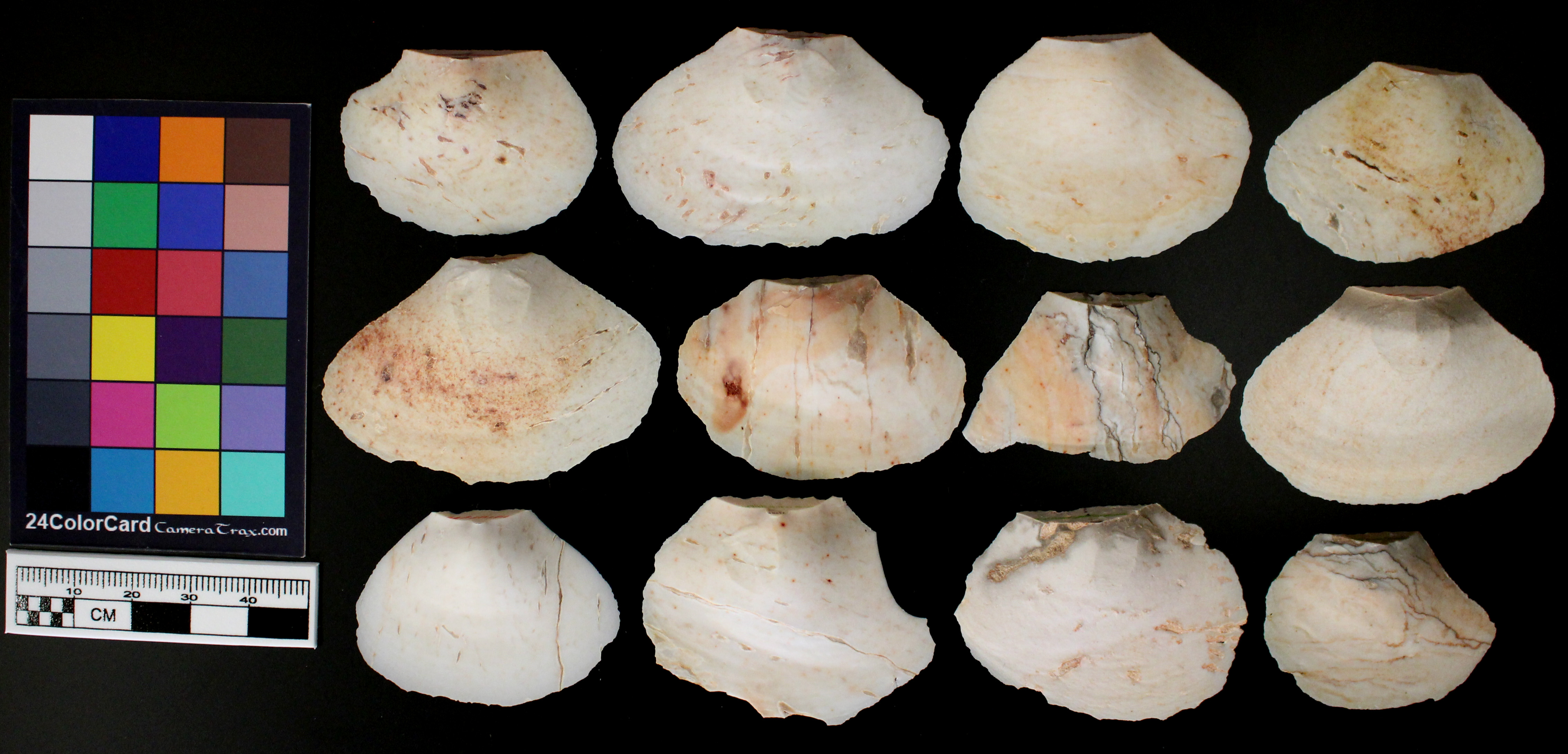


Fig. S4. Photograph of select flakes removed from cores heated to 350ºC.

1. **Results of General Linear Modeling of flake form variables**

We used a general linear modeling (GLM) procedure to examine each of the seven variables measuring the form of the experimentally-derived flakes (Weight, Length, Width, Width at 25%, Width at 50%, Width at 75%, and Thickness) while controlling for force magnitude, exterior platform angle, and platform depth by temperature group (ambient or raw, heated to 300ºC, and heated to 350ºC). The results indicate that none of the variables measuring flake form are significant when force magnitude, exterior platform, and platform depth are controlled in a GLM. Individual GLM results are presented below:

**Model 1 for weight:**

glm(formula = Wg ~ Force + EPA * PD * Temperature)

**Coefficients:**

|  | **Estimate** | **Std. Error** | **t-value** | **p-value** |
| --- | --- | --- | --- | --- |
| (Intercept) | 21.1875 | 33.8943 | 0.625 | 0.534 |
| Force | 0.0004 | 0.0003 | 1.330 | 0.188 |
| EPA | -0.3880 | 0.5428 | -0.715 | 0.477 |
| PD | -6.9733 | 6.5457 | -1.065 | 0.290 |
| Temperature1 | 7.7593 | 52.0705 | 0.149 | 0.882 |
| Temperature2 | -48.3341 | 43.9057 | -1.101 | 0.274 |
| EPA:PD | 0.1354 | 0.1053 | 1.286 | 0.202 |
| EPA:Temperature1 | -0.1023 | 0.8270 | -0.124 | 0.902 |
| EPA:Temperature2 | 0.7649 | 0.7087 | 1.079 | 0.284 |
| PD:Temperature1 | -1.9616 | 9.9056 | -0.198 | 0.844 |
| PD:Temperature2 | 9.5575 | 8.6713 | 1.102 | 0.274 |
| EPA:PD:Temperature1 | 0.0265 | 0.1573 | 0.169 | 0.866 |
| EPA:PD:Temperature2 | -0.1503 | 0.1404 | -1.071 | 0.288 |

**Model 2 for length:**

glm(formula = L ~ Force + EPA * PD * Temperature)

**Coefficients:**

|  | **Estimate** | **Std. Error** | **t-value** | **p-value** |
| --- | --- | --- | --- | --- |
| (Intercept) | 93.4108 | 114.354 | 0.817 | 0.417 |
| Force | 0.0000 | 0.001 | 0.028 | 0.978 |
| EPA | -1.2543 | 1.831 | -0.685 | 0.495 |
| PD | -22.2402 | 22.084 | -1.007 | 0.317 |
| Temperature1 | -90.0580 | 175.677 | -0.513 | 0.610 |
| Temperature2 | -41.7830 | 148.131 | -0.282 | 0.779 |
| EPA:PD | 0.4118 | 0.355 | 1.159 | 0.250 |
| EPA:Temperature1 | 1.5859 | 2.790 | 0.568 | 0.571 |
| EPA:Temperature2 | 0.5419 | 2.391 | 0.227 | 0.821 |
| PD:Temperature1 | 15.9449 | 33.420 | 0.477 | 0.635 |
| PD:Temperature2 | 2.6634 | 29.255 | 0.091 | 0.928 |
| EPA:PD:Temperature1 | -0.2837 | 0.531 | -0.534 | 0.595 |
| EPA:PD:Temperature2 | -0.0124 | 0.474 | -0.026 | 0.979 |

**Model 3 for width:**

glm(formula = W ~ Force + EPA * PD * Temperature)

**Coefficients:**

|  | **Estimate** | **Std. Error** | **t-value** | **p-value** |
| --- | --- | --- | --- | --- |
| (Intercept) | 124.408 | 164.686 | 0.755 | 0.452 |
| Force | 0.001 | 0.001 | 0.651 | 0.517 |
| EPA | -1.671 | 2.638 | -0.634 | 0.528 |
| PD | -33.579 | 31.804 | -1.056 | 0.294 |
| Temperature1 | 104.784 | 253.001 | 0.414 | 0.680 |
| Temperature2 | -274.796 | 213.330 | -1.288 | 0.202 |
| EPA:PD | 0.619 | 0.512 | 1.210 | 0.230 |
| EPA:Temperature1 | -1.557 | 4.018 | -0.388 | 0.699 |
| EPA:Temperature2 | 4.261 | 3.443 | 1.237 | 0.220 |
| PD:Temperature1 | -21.734 | 48.129 | -0.452 | 0.653 |
| PD:Temperature2 | 50.542 | 42.132 | 1.200 | 0.234 |
| EPA:PD:Temperature1 | 0.322 | 0.764 | 0.421 | 0.675 |
| EPA:PD:Temperature2 | -0.780 | 0.682 | -1.144 | 0.256 |

**Model 4 for width at 25%:**

glm(formula = W25 ~ Force + EPA * PD * Temperature)

**Coefficients:**

|  | **Estimate** | **Std. Error** | **t-value** | **p-value** |
| --- | --- | --- | --- | --- |
| (Intercept) | 93.391 | 115.021 | 0.812 | 0.419 |
| Force | 0.001 | 0.001 | 1.112 | 0.269 |
| EPA | -1.255 | 1.842 | -0.681 | 0.498 |
| PD | -20.793 | 22.213 | -0.936 | 0.352 |
| Temperature1 | 79.972 | 176.703 | 0.453 | 0.652 |
| Temperature2 | -229.980 | 148.995 | -1.544 | 0.127 |
| EPA:PD | 0.401 | 0.357 | 1.123 | 0.265 |
| EPA:Temperature1 | -1.126 | 2.807 | -0.401 | 0.689 |
| EPA:Temperature2 | 3.631 | 2.405 | 1.510 | 0.135 |
| PD:Temperature1 | -14.040 | 33.615 | -0.418 | 0.677 |
| PD:Temperature2 | 35.790 | 29.426 | 1.216 | 0.228 |
| EPA:PD:Temperature1 | 0.196 | 0.534 | 0.368 | 0.714 |
| EPA:PD:Temperature2 | -0.563 | 0.476 | -1.183 | 0.241 |

**Model 5 for width at 50%:**

glm(formula = W50 ~ Force + EPA * PD * Temperature)

**Coefficients:**

|  | **Estimate** | **Std. Error** | **t-value** | **p-value** |
| --- | --- | --- | --- | --- |
| (Intercept) | 128.559 | 164.355 | 0.782 | 0.436 |
| Force | 0.001 | 0.001 | 0.742 | 0.460 |
| EPA | -1.742 | 2.632 | -0.662 | 0.510 |
| PD | -34.737 | 31.740 | -1.094 | 0.277 |
| Temperature1 | 79.578 | 252.492 | 0.315 | 0.753 |
| Temperature2 | -244.055 | 212.901 | -1.146 | 0.255 |
| EPA:PD | 0.636 | 0.511 | 1.247 | 0.216 |
| EPA:Temperature1 | -1.171 | 4.010 | -0.292 | 0.771 |
| EPA:Temperature2 | 3.763 | 3.436 | 1.095 | 0.277 |
| PD:Temperature1 | -16.813 | 48.033 | -0.350 | 0.727 |
| PD:Temperature2 | 43.196 | 42.047 | 1.027 | 0.307 |
| EPA:PD:Temperature1 | 0.247 | 0.763 | 0.323 | 0.747 |
| EPA:PD:Temperature2 | -0.661 | 0.681 | -0.971 | 0.334 |

**Model 6 for width at 75%:**

glm(formula = W75 ~ Force + EPA * PD * Temperature)

**Coefficients:**

|  | **Estimate** | **Std. Error** | **t-value** | **p-value** |
| --- | --- | --- | --- | --- |
| (Intercept) | 131.513 | 168.866 | 0.779 | 0.438 |
| Force | 0.001 | 0.001 | 1.032 | 0.305 |
| EPA | -1.853 | 2.704 | -0.685 | 0.495 |
| PD | -34.747 | 32.611 | -1.065 | 0.290 |
| Temperature1 | 85.302 | 259.423 | 0.329 | 0.743 |
| Temperature2 | -213.642 | 218.745 | -0.977 | 0.332 |
| EPA:PD | 0.626 | 0.525 | 1.192 | 0.237 |
| EPA:Temperature1 | -1.306 | 4.120 | -0.317 | 0.752 |
| EPA:Temperature2 | 3.269 | 3.531 | 0.926 | 0.357 |
| PD:Temperature1 | -16.743 | 49.351 | -0.339 | 0.735 |
| PD:Temperature2 | 42.041 | 43.201 | 0.973 | 0.334 |
| EPA:PD:Temperature1 | 0.252 | 0.784 | 0.321 | 0.749 |
| EPA:PD:Temperature2 | -0.639 | 0.699 | -0.914 | 0.363 |

**Model 7 for width at Th:**

glm(formula = Th ~ Force + EPA * PD * Temperature)

**Coefficients:**

|  | **Estimate** | **Std. Error** | **t-value** | **p-value** |
| --- | --- | --- | --- | --- |
| (Intercept) | 9.4808 | 40.7766 | 0.233 | 0.817 |
| Force | -0.0000 | 0.0003 | -0.132 | 0.895 |
| EPA | -0.1330 | 0.6531 | -0.204 | 0.839 |
| PD | -0.7739 | 7.8748 | -0.098 | 0.922 |
| Temperature1 | 4.4627 | 62.6435 | 0.071 | 0.943 |
| Temperature2 | -26.2299 | 52.8209 | -0.497 | 0.621 |
| EPA:PD | 0.0265 | 0.1267 | 0.209 | 0.835 |
| EPA:Temperature1 | -0.0542 | 0.9950 | -0.054 | 0.957 |
| EPA:Temperature2 | 0.4147 | 0.8526 | 0.486 | 0.628 |
| PD:Temperature1 | -1.6362 | 11.9169 | -0.137 | 0.891 |
| PD:Temperature2 | 4.0425 | 10.4320 | 0.388 | 0.699 |
| EPA:PD:Temperature1 | 0.0223 | 0.1893 | 0.118 | 0.906 |
| EPA:PD:Temperature2 | -0.0636 | 0.1689 | -0.377 | 0.707 |

1. Data on cores (CSV file: ‘Data on experimental cores.csv’)
2. Data on force and flake measurements (CSV file: ‘Instron force and flake data.csv’)
3. R script for analyses (R file:’Instrondata.R’)
